# Protective and Risk Factors of Depression Among Older Adults During the COVID-19 Pandemic

**DOI:** 10.1093/geroni/igab046.2688

**Published:** 2021-12-17

**Authors:** Yeonjung Lee, Tyran Terada

**Affiliations:** 1 University of Hawaiʻi at Mānoa, Honolulu, Hawaii, United States; 2 University of Hawai`i, Honolulu, Hawaii, United States

## Abstract

The coronavirus disease 2019 (COVID-19) pandemic adversely impacted the mental health of older adults. This study aims to explore the associations between protective/risk factors of depression during the pandemic and to examine the differences in these associations by marital status. Data from the Health and Retirement Study 2020 COVID-19 module, released in February, 2021, were used. The level of resilience during the pandemic was selected as a protective factor. The level of COVID-19 pandemic concern was selected as a risk factor. Among older adults aged 51 years and older, the weighted regression model found that higher levels of COVID-19 concern were associated with higher levels of depressive symptoms (p<0.05), whereas higher levels of resilience were associated with lower levels of depressive symptoms (p<0.05). Marital status moderated the association between COVID-19 concern and depressive symptoms. Never-married people were at higher risk of depressive symptoms than married people when COVID-19 concerns increased. It is important to enhance support for never-married people during the pandemic to protect their psychological well-being.

